# Enteroptosis

**Published:** 1902-10-11

**Authors:** 


### ENTEROPTOSIS.

In a paper dealing with enteroptosis combined, as is usually the case, with nephroptosis, Dr. Forbes Hawkes,[1] of New York, suggests that this condition may in many cases be started in infancy, being caused by the repeated more or less sudden motions conveyed to the child by the nurse or mother at a time when the very delicate and elastic intra-abdominal tissues of the infant are in an extremely favourable condition to be stretched, the gastro-intestinal tract being more or less filled all the time with the bulky food of the first year of the child's life. The comparatively large size of the liver at the time would, he says, determine the descent of the right kidney at this early period.

Coming to treatment, the novelty lies in the suggestion of a prolonged partial inversion of the patient after a lumbar nephrorrhaphy. After the operation, Dr. Hawkes keeps the patient for five or six weeks lying on a bed, the foot of which is raised 18 inches from the floor. After that time is passed, the foot of the bed is lowered 3 or 4 inches a day, until the horizontal is reached, when the upright position is gradually assumed. Later, a tight straight-front corset is ordered, and this is worn for three months whenever the patient is on her feet, being applied before rising, and removed after getting into bed. In addition to this, lying down for an hour during the day, with the head low and the hips high, is advised, with exercises to strengthen the lateral and anterior abdominal muscles, and out-door exercises such as golf, walking, and moderate tennis, but not horse-back riding. How far the inversion, the "straight-front corset," the rest, and the exercises would do without the nephrorrhaphy we are not told.

[1] The Post Graduate, Sept., 1902.

